# Role of Two Metacaspases in Development and Pathogenicity of the Rice Blast Fungus Magnaporthe oryzae

**DOI:** 10.1128/mBio.03471-20

**Published:** 2021-02-09

**Authors:** Jessie Fernandez, Victor Lopez, Lisa Kinch, Mariel A. Pfeifer, Hillery Gray, Nalleli Garcia, Nick V. Grishin, Chang-Hyun Khang, Kim Orth

**Affiliations:** a Department of Molecular Biology, University of Texas Southwestern Medical Center, Dallas, Texas, USA; b Department of Biophysics and Biochemistry, University of Texas Southwestern Medical Center, Dallas, Texas, USA; c Howard Hughes Medical Institute (HHMI), Dallas, Texas, USA; d Department of Plant Biology, University of Georgia, Athens, Georgia, USA; e Department of Biochemistry, University of Texas Southwestern Medical Center, Dallas, Texas, USA; Universidad de Córdoba

**Keywords:** *Magnaporthe oryzae*, rice blast, disease, metacaspase, pathogenesis

## Abstract

Rice blast disease caused by Magnaporthe oryzae is a devastating disease of cultivated rice worldwide. Infections by this fungus lead to a significant reduction in rice yields and threats to food security. To gain better insight into growth and cell death in *M. oryzae* during infection, we characterized two predicted *M. oryzae* metacaspase proteins, MoMca1 and MoMca2. These proteins appear to be functionally redundant and can complement the yeast Yca1 homologue. Biochemical analysis revealed that *M. oryzae* metacaspases exhibited Ca^2+^-dependent caspase activity *in vitro*. Deletion of both *MoMca1* and *MoMca2* in *M. oryzae* resulted in reduced sporulation, delay in conidial germination, and attenuation of disease severity. In addition, the double Δ*Momca1mca2* mutant strain showed increased radial growth in the presence of oxidative stress. Interestingly, the Δ*Momca1mca2* strain showed an increased accumulation of insoluble aggregates compared to the wild-type strain during vegetative growth. Our findings suggest that MoMca1 and MoMca2 promote the clearance of insoluble aggregates in *M. oryzae*, demonstrating the important role these metacaspases have in fungal protein homeostasis. Furthermore, these metacaspase proteins may play additional roles, like in regulating stress responses, that would help maintain the fitness of fungal cells required for host infection.

## Abstract (precis)

Magnaporthe oryzae causes rice blast disease that threatens global food security by resulting in the severe loss of rice production every year. A tightly regulated life cycle allows *M. oryzae* to disarm the host plant immune system during its biotrophic stage before triggering plant cell death in its necrotrophic stage.

## INTRODUCTION

Caspases are a family of conserved cysteine-dependent, aspartate-specific proteases that play an essential role in metazoan programmed cell death. They regulate multiple cellular behaviors that contribute to organism fitness and pathology (1). This family of proteases contains a unique α/β hemoglobinase fold that consists of a large subunit (p20) containing the catalytic histidine/cysteine dyad and a small subunit (p10) (2). Caspases are synthesized as zymogens, and, upon an external stimulus, are activated by autocatalysis. At present, there are no known caspase homologs in nonmetazoan organisms; however, a group of distantly related orthologous caspases known as metacaspases was discovered in fungi, plant, and protozoa (2–5). Metacaspases share structural homology to components of animal caspases but lack substrate specificity for aspartate residues (3). Instead, they specifically cleave substrates preceded by positively charged lysine and arginine residues in the P1 position (3, 5). Bioinformatics analysis identified three types of metacaspases based on the presence or absence of an N-terminal prodomain with the caspase domain organization (p20/p10 subunits) (2). For instance, the budding yeast Saccharomyces cerevisiae harbors a single type I metacaspase known as Yca1 (4). Similar to animal caspases, Yca1 contains the conserved Cys-His catalytic dyad and undergoes autoproteolytic processing to yield p20 (∼20-kDa) and p10 (∼12-kDa) subunits from the inactive zymogen (4). The N-terminal prodomain is rich on poly-Q/N repeats, a motif predicted to be involved in self-aggregation (6).

Overall, metacaspases are multifunctional proteases essential for normal physiology of nonmetazoan groups. For instance, the loss or inactivation of Yca1 in yeast alters the timing of the cell cycle progression by elongating the G_1_ phase and perturbing the G_2_/M mitotic checkpoint, implicating Yca1 in the regulation of the cell cycle (7). Moreover, Yca1 stimulates apoptotic-like cell death during oxidative stress and aging in yeast (4, 8–11). In the absence of this caspase, yeast cells tolerate low doses of H_2_O_2_, promoting their survival under these harsh conditions (4). Yca1 has also been implicated in the clearance of insoluble protein aggregates, thereby protecting cells against by-products of aging and toxic amyloids (6). Studies demonstrated that Yca1 interacts with known members of the proteostasis network, such as Cdc48, Hsp104, and the Hsp70/40 chaperone systems (6). Indeed, the loss of Yca1 results in the accumulation of stress response chaperones, leading to the enrichment of autophagic bodies as a compensatory response of survival (6). Further investigations established that Yca1 maintains proteostasis through direct interaction with the ubiquitin proteasome system (12). Specifically, the ubiquitination of Yca1 was shown to have a direct impact on its function within the proteostasis network; once Yca1 was ubiquitinated, the yeast was able to regulate protein aggregation levels and autophagy (12). Metacaspases have also been discovered in a few other fungus groups, like *Aspergillus* spp., Podospora anserina, Candida albicans, and, recently, in the corn smut Ustilago maydis (13–17). Like Yca1 in yeast, most of these fungal caspases have been linked to stress- and age-related responses as consequences of several environmental stimuli (13–17).

The filamentous fungus Magnaporthe oryzae is the causative agent of rice blast, one of the most destructive diseases of cultivated rice in the world, and it is responsible for the annual destruction of approximately 10 to 30% of the rice harvested globally (18). During infection, *M. oryzae* undergoes extensive developmental changes, which allow it to break into plant cells, build elaborate infection structures, and proliferate inside host cells without causing visible disease symptoms (18). The infection starts when the three-celled conidium attaches to the surface of the rice leaf. The conidium germinates and forms a germ tube. The tip of the germ tube differentiates into an infection structure called the appressorium (19, 20). Once the appressorium matures, turgor pressure builds within the structure and drives the protrusion of the penetration peg, enabling it to breach the leaf cuticle (18, 21). Subsequently, *M. oryzae* colonizes the plant tissue, sporulates, and spreads by air to uninfected rice plants to continue its life cycle.

Here, we identify and characterize two *M. oryzae* metacaspases, MoMca1 and MoMca2, which exhibit a C14 peptidase activity. While the Cys/His catalytic dyad is essential for its autocatalytic processing, these metacaspases require Ca^2+^ for their full proteolytic activity *in vitro*, suggesting a critical role *in vivo* for this catalytic mechanism. In the absence of both *MoMca1* and *MoMca2*, *M. oryzae* exhibits delayed conidial germination and subsequent delayed appressorium formation. Moreover, the double mutant strain, but not the single mutant strains, was impaired in developing typical wild-type (WT) lesions on rice leaves compared to the WT strain, suggesting that MoMca1 and MoMca2 are redundant and that both are required for full pathogenicity. Consistent with this observation, both enzymes can functionally complement the yeast Δ*yca1* strain. Interestingly, we observed that these proteins promote the clearance of insoluble aggregates to maintain the fitness of *M. oryzae* cells. Collectively, our study demonstrates that the *MoMca* genes play important roles in growth, conidiation, appressorium development, and pathogenesis for *M. oryzae*.

## RESULTS

### *M. oryzae* genome contains two putative metacaspase proteins.

**FIG 1:**
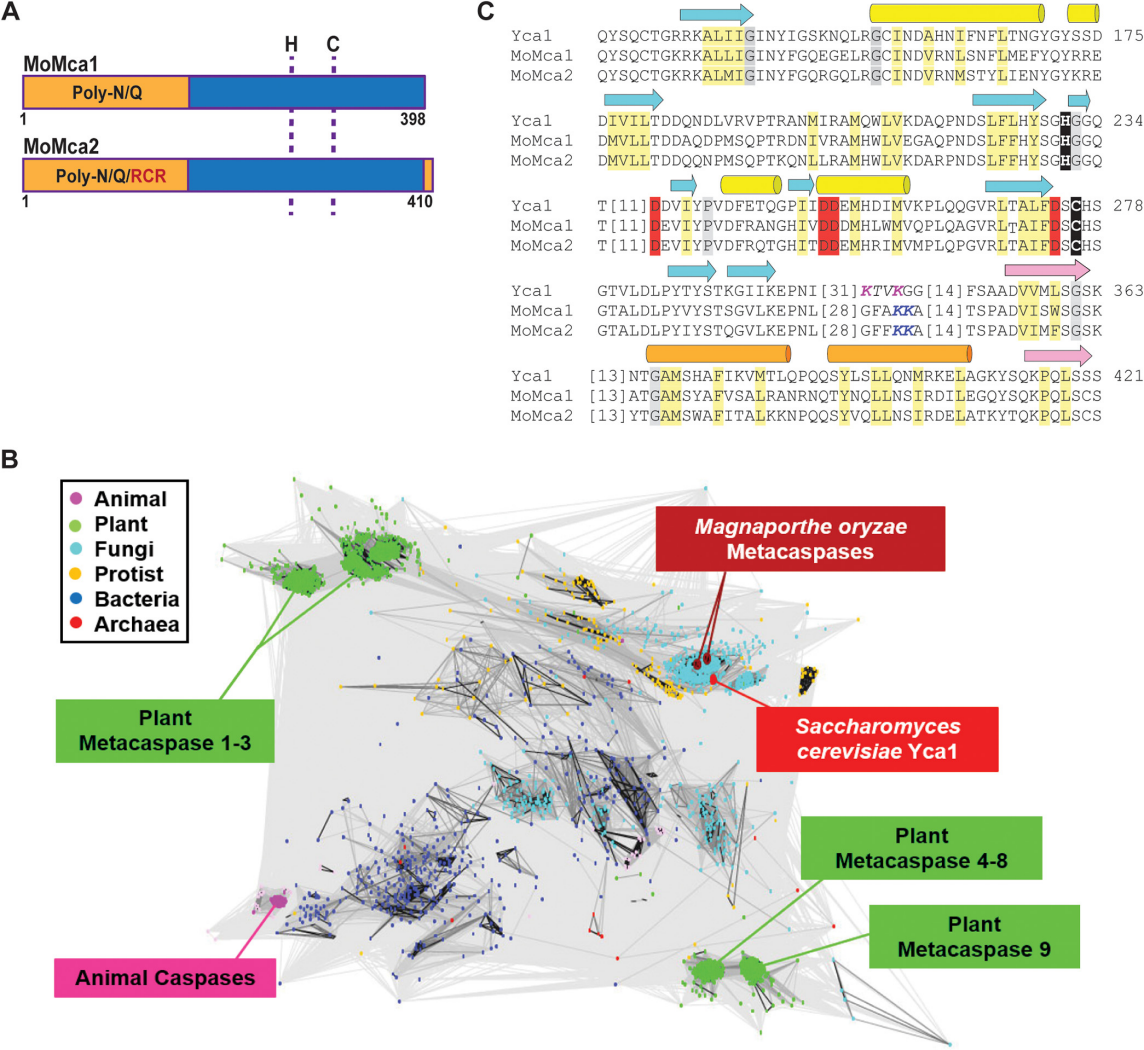
Bioinformatics identification of two Magnaporthe oryzae metacaspases. (A) Schematic representation of MoMca1 (MGG_04926) and MoMca2 (MGG_13530). The prodomain, rich in asparagine (N) and glutamine (Q) residues, is indicated in orange. The caspase domain is marked in blue. RCR domain, partial sequence motif of chitin synthesis regulation, labeled in red. Dashed lines show the position of the histidine (H) and cysteine (C) catalytic dyad. (B) CLANS clustering analysis is represented graphically as the network of BLAST-derived sequence similarities between 2,712 metacaspase protein sequences. Dots represent sequences, with the shade of connecting lines denoting similarity from gray (low) to black (high). (C) The catalytic sites of MoMca1 and MoMca2 are aligned with Yca1, highlighting conserved active-site residues (black), calcium binding residues (red), Yca1 cleavage site (magenta), predicted MoMca1/MoMca2 cleavage site (Blue), mainly hydrophobic positions (light yellow), and mainly small positions (gray). Beta-strands (arrow) and alpha-helices (cylinder) from the Yca1 structure are indicated above the alignment and colored cyan/yellow before and pink/orange after the cleavage site (italics).

To investigate critical roles of metacaspases in the *M. oryzae* life cycle, we first searched the *Magnaporthe* genome for metacaspase-related genes. We chose to analyze this group of proteins because they have been extensively studied in yeast but remain largely uncharacterized in filamentous, plant-pathogenic fungi. Using the protein sequence of yeast Yca1 for BLASTP searches, we identified two putative metacaspase genes, MGG_04626 and MGG_13530 (now named *MoMca1* and *MoMca2*, respectively), in the *M. oryzae* genome database. *MoMca1* and *MoMca2* genes are located on chromosomes Chr3 and Chr4, respectively. These putative proteases are predicted to encode a typical type I metacaspase containing an N-terminal prodomain with a Q/N-rich repeat motif and a C-terminal peptidase-C14 caspase domain (Fig. 1A). MoMca1 and MoMca2 encode 396- and 410-amino-acid proteins, respectively. Clan clustering analysis of 2,712 metacaspase protein sequences showed that *M. oryzae* metacaspases clustered in the same clan as yeast Yca1, with their catalytic domains sharing approximately 45% sequence similarity (Fig. 1B). Bioinformatics analysis also revealed MoMca1 and MoMca2 share 67% similarity between their catalytic domains. Using ClustalW, the sequence alignment of MoMca1 and MoMca2 with Yca1 illustrated that *M. oryzae* proteins contain the predicted histidine and cysteine as the catalytic dyad specific for clan CD proteases (Fig. 1A and C). In Fig. S1A in the supplemental material, the tertiary structure of full-length *M. oryzae* metacaspases is modeled using the structural template of yeast Yca1 according to Swiss-Model (PDB entry 4F6P) (22).

10.1128/mBio.03471-20.1 FIG S1 (A) The structure (PDB entry 4F6O) is colored the same as the alignment, with functional residues shown in sticks. The cleavage site is disordered, shown by a dashed line (magenta). The calcium is represented by a samarium ion from a superimposed homolog (PDB entry 4AFP). (B) Split marker method diagram. SP stands for selectable primer. *ILV1* and *HPH* genes confer resistance against sulfonylurea drug and hygromycin selection, respectively. (C) Deleted mutant strains were confirmed by PCR. Primers P1 and P4 amplified 1 kb upstream and downstream for the *MoMca1* and *MoMca2* genes. Top gels, left, *MoMca1*; right, *MoMca2*. Primers P5 and P6 amplified internal sequences of *Momca1* and *Momca2* genes. Metacaspase genes in the WT genome contain several introns. Bottom gels, left, *MoMca1*; right, *MoMca2*. The *Momca1* gene was replaced in the Δ*Momca1mca2* mutant with the *hph* gene that confers resistance to hygromycin selection. The complemented strain, Δ*Momca1mca2-C*, was generated by cloning the *MoMca1* and *MoMca2* ORFs with their respective 1-kb native promoters into the binary pBHt2 vector and transfected into Δ*Momca1mca2* protoplasts. Download FIG S1, TIF file, 1.3 MB. Copyright © 2021 Fernandez et al. 2021 Fernandez et al. This content is distributed under the terms of the Creative Commons Attribution 4.0 International license.

### MoMca1 and MoMca2 are Ca^2+^-dependent proteases.

**FIG 2:**
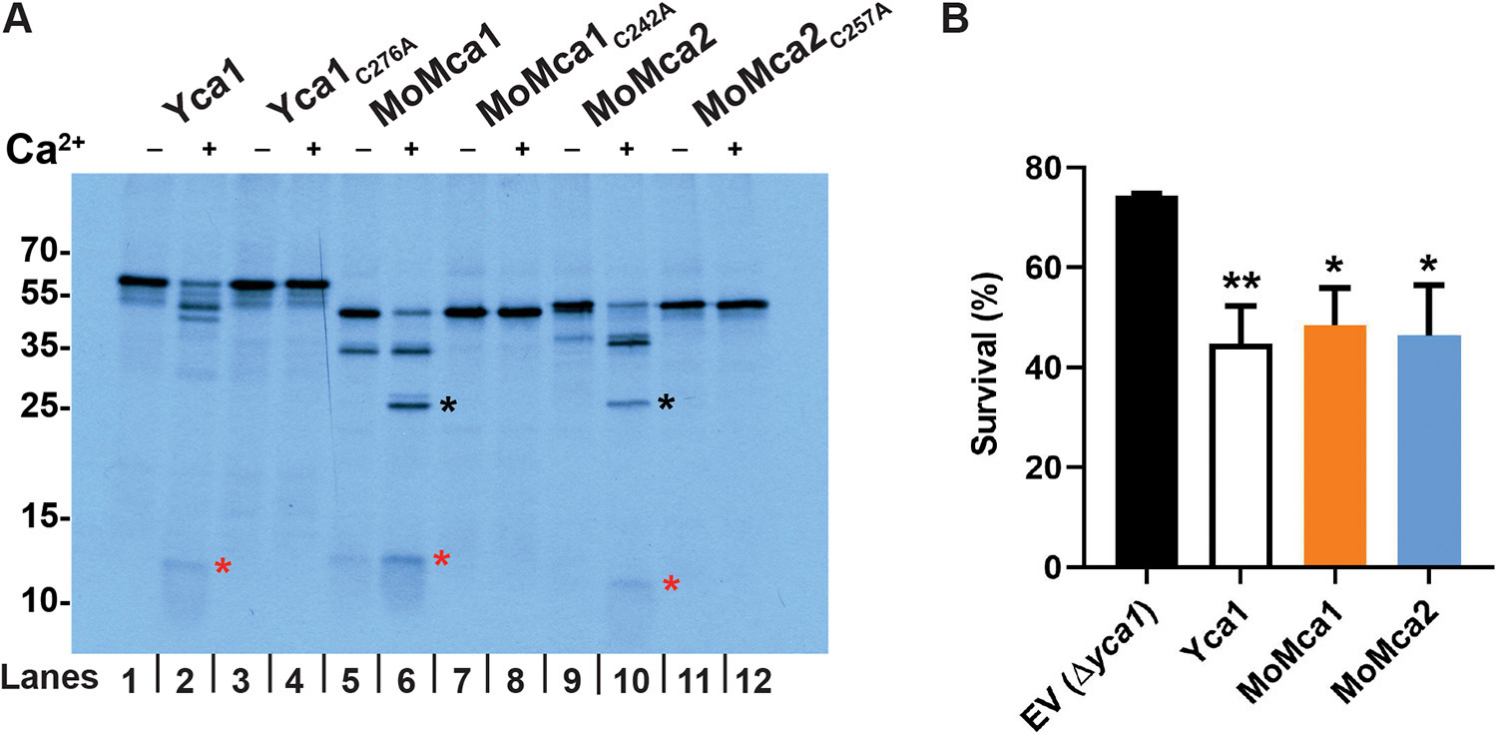
Biochemical characterization of Magnaporthe oryzae metacaspases. (A) Protease activity assay of MoMca1 and MoMca2 in the presence or absence of 1 mM CaCl_2_ *in vitro*. Red and black asterisks indicate the p20 and p10 subunits, respectively. Samples were loaded in pairs; odd lanes are controls for even lanes. (B) Survival assay of Δ*yca1* cells expressing Yca1, MoMca1, MoMca2, and the corresponding empty vector control (EV Δ*yca1*) after 24 h of galactose induction, shown as percent survival in the presence of 1.2 mM H_2_O_2_ treatment. Data are represented as the means from three independent measurements. Error bars denote standard deviations. Asterisks indicate statistically significant differences (*, *P* < 0.05; **, *P* < 0.01; ***, *P* < 0.001; one-way ANOVA with Tukey’s multiple-comparison test using GraphPad Prism 8).

For most of the metacaspases studied so far, autocatalysis of an inactive zymogen is responsible for generating an active enzyme monomer (4, 5, 22). Therefore, we addressed the question of whether MoMca1 and MoMca2 undergo autocatalytic activation in a manner similar to that of yeast Yca1. Due to the insolubility of both *M. oryzae* metacaspase proteins in bacterial cells, we decided to use an *in vitro* transcription/translation approach to enable the rapid expression of the full-length proteins MoMca1 and MoMca2. A recent study demonstrated that *Arabidopsis* metacaspase AtMCP2d and yeast Yca1 strictly require Ca^2+^ for their proteolytic activity *in vitro* (5). We observed that recombinant MoMca1 and MoMca2 are already autocatalytically processed upon translation, yielding an ∼35-kDa intermediate common to both metacaspases and the p10 subunit, at ∼12 kDa in MoMca1 and ∼10 kDa in MoMca2 (Fig. 2A). To examine whether treatment with divalent cations such as Ca^2+^ positively affects *M. oryzae* metacaspase autoprocessing, we added 1 mM CaCl_2_ to the translation mixture and observed further autocatalysis, yielding an ∼25-kDa fragment of the large subunit (Fig. 2A). These results demonstrate that Ca^2+^ specifically enhanced the autocatalytic processing of MoMca1 and MoMca2.

Sequence alignment of MoMca1 and MoMca2 with Yca1 showed the predicted catalytic Cys/His dyad residues in both *M. oryzae* metacaspases (Fig. 1C). The catalytic cysteine (Cys^242^ for MoMca1 and Cys^257^ for MoMca2) was replaced by an alanine residue in their coding sequences. Figure 2A shows that in the presence of Ca^2+^, the catalytic dead proteins are unable to autoprocess and generate catalytic subunits, remaining inactive zymogens. These results demonstrated that the predicted conserved cysteines are essential for metacaspase autoproteolysis.

### Expression of MoMca1 and MoMca2 in a heterologous system.

Previous studies demonstrated that low doses of H_2_O_2_ promote the expression of Yca1 in yeast, leading to the induction of apoptosis (4). The Δ*yca1* deletion strain exhibits higher rates of survival under oxidative stress conditions than the wild-type strain (4). To determine if MoMca1 and MoMca2 functionally complement Yca1 in H_2_O_2_-mediated apoptosis, we cloned the *M. oryzae* metacaspases into the Δ*yca1* yeast strain and grew these strains in liquid galactose inducing medium containing 1.2 mM H_2_O_2_ for 24 h. Figure 2B shows a significant reduction in cell survival observed upon complementation of the Δ*yca1* strain containing either *MoMca1* or *MoMca2* compared to the Δ*yca1* strain carrying the empty vector. These results indicate that MoMca1 and MoMca2 can functionally complement Yca1 when expressed in S. cerevisiae.

### MoMca1 and MoMca2 are required for the virulence of *M. oryzae*.

**FIG 3:**
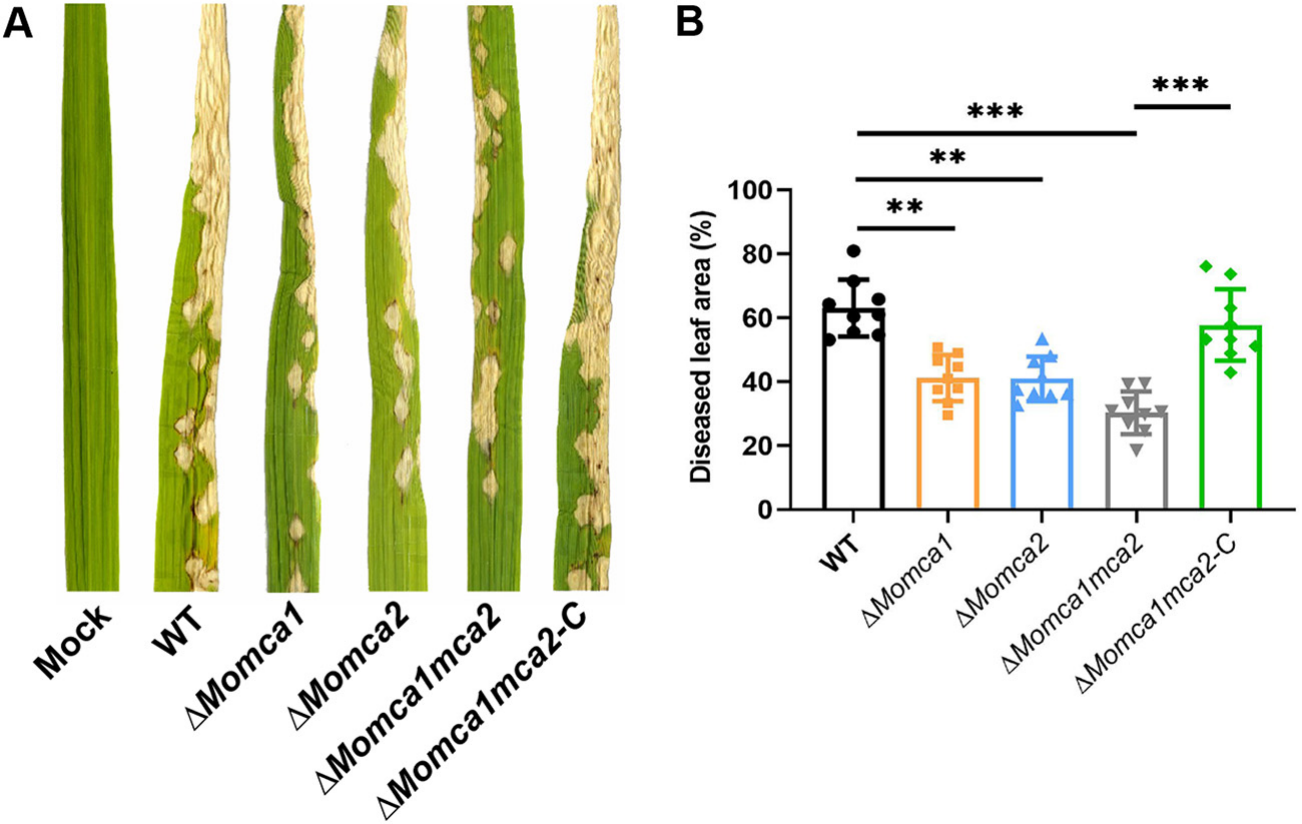
*MoMca1* and *MoMca2* are required for full pathogenicity. (A and B) Δ*Momca1mca2* strain was reduced in virulence compared to that of the WT when applied to leaves of the susceptible rice variety YT16. Δ*Momca1mca2*-*C* refers to the complemented strain. (B) Diseased leaf area was measured using ImageJ. Results are the means from three independent measurements. Error bars denote standard deviations. Asterisks indicate statistically significant differences (**, *P* < 0.01; ***, *P* < 0.001; one-way ANOVA with Tukey’s multiple-comparison test using GraphPad Prism 8).

To determine if *MoMca1* and *MoMca2* play a role in *M. oryzae* pathogenicity, we generated single Δ*Momca1* and Δ*Momca2* deletion strains, a double mutant strain, Δ*Momca1mca2*, and a complemented strain, Δ*Momca1mca2-C*, containing pBGt MoMCA1MCA2 expression vector (Fig. S1B and C). These strains were evaluated for pathogenicity on rice leaves. We inoculated susceptible rice plants with spore suspensions of the wild-type (WT) or mutant strains. The WT strain exhibited the expected necrotic lesions on rice leaves. In contrast, the Δ*Momca1mca2* strain displayed a significant reduction in disease severity compared to the WT and the single mutants. (Fig. 3A and B). These data suggest that metacaspases are required for full pathogenicity in rice blast fungus, signifying their importance in *M. oryzae* pathogenicity.

### *M. oryzae* metacaspases are required for sporulation.

To investigate the role of MoMca1 and MoMca2 in growth and development of *M. oryzae*, we assessed the WT, mutant, and complemented strains for colony diameter on complete medium (CM; Fig. S2A and B). However, the Δ*Momca1mca2* strain displayed severely reduced sporulation compared to the WT and single mutant strains (Fig. S2C). Moreover, none of these strains showed differences in sensitivity to cell wall assembly inhibitor or osmotic stress (Fig. S3A and B). Taken together, these data suggest that metacaspases are individually dispensable for spore development and appropriate vegetative growth under axenic conditions and are redundant in providing an essential function for spore development.

10.1128/mBio.03471-20.2 FIG S2 (A and B) Radial growth on complete medium (CM) was not impaired in mutant strains carrying gene deletions in *MoMca1*, *MoMca2*, or the double mutant. KV1 is the wild-type (WT) isolate used in this study. Images were taken at 5 days after incubation. (C) Sporulation was impaired in strains lacking functional *Mca* genes but more significantly reduced in the Δ*Momca1mca2* strain during growth on CM. Values represent means from three independent replicates. Error bars denote standard deviations. Asterisks indicate statistically significant results: ***, *P < *0.001 (one-way ANOVA with Tukey’s multiple-comparison test using GraphPad Prism 8). Download FIG S2, TIF file, 1 MB. Copyright © 2021 Fernandez et al. 2021 Fernandez et al. This content is distributed under the terms of the Creative Commons Attribution 4.0 International license.

10.1128/mBio.03471-20.3 FIG S3 Metacaspase Δ*Momca1*, Δ*Momca2*, and Δ*Momca1mca2* mutants are not sensitive to the osmolytes 1 M sorbitol and 1 M NaCl and cell wall assembly inhibitor Congo Red. (A) Stressors were added to CM at the concentrations shown. Images were taken after 5 days of growth. (B) Relative density of the aggregates on insoluble fractions in the spores and mycelial lysates. The relative density was measured using ImageJ. The relative values were obtained from three independent experiments with technical repetitions. Error bars denote SEM. Asterisks indicate statistically significant differences (**, *P < *0.01; ****, *P < *0.0001; one-way ANOVA with Tukey’s multiple-comparison test using GraphPad Prism 8). Download FIG S3, TIF file, 2.2 MB. Copyright © 2021 Fernandez et al. 2021 Fernandez et al. This content is distributed under the terms of the Creative Commons Attribution 4.0 International license.

### MoMca1 and MoMca2 are expressed under oxidative conditions *in vivo*.

**FIG 4:**
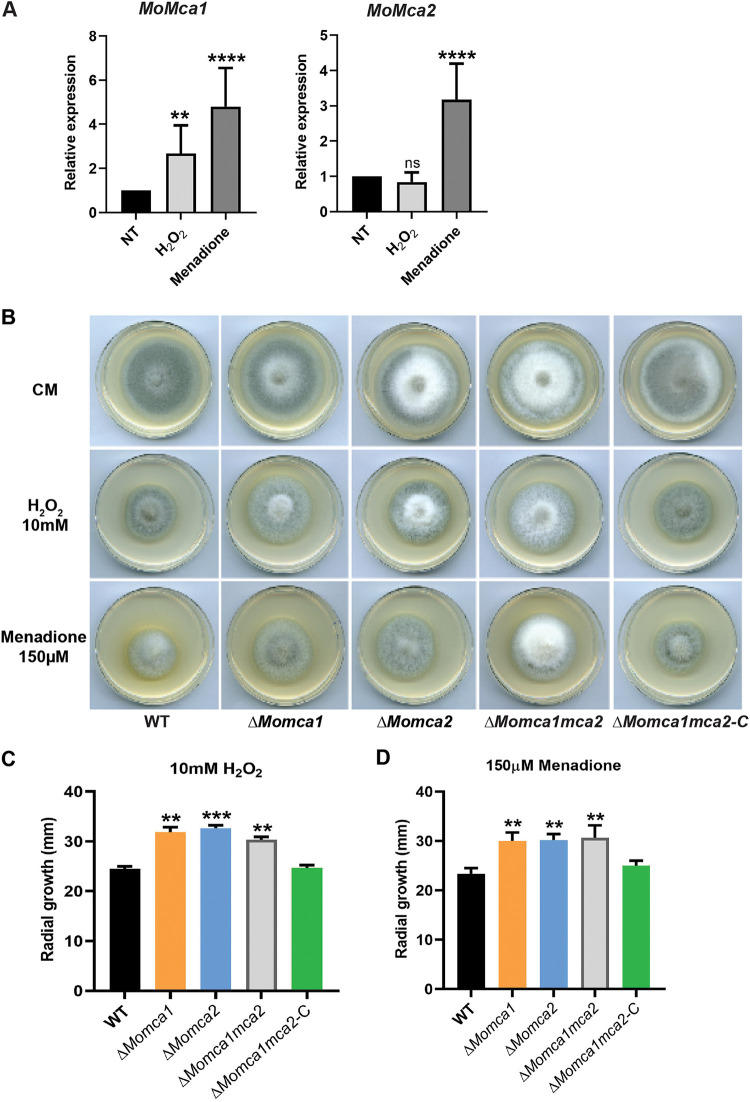
Δ*Momca1*, Δ*Momca2*, and Δ*Momca1mca2* mutant strains showed resistance to the oxidative stress conditions compared to the WT strain. (A) Quantitative RT-PCR analysis showing the expression of *M. oryzae* metacaspase-encoding genes, *MoMca1* and *MoMca2*, in the WT KV1 strain under oxidative stress conditions. Fungal mycelia were grown in liquid CM for 48 h at 25°C with agitation (150 rpm) before treatment with either 5 mM H_2_O_2_ or 100 μM menadione for 1 h. Expression was normalized to the housekeeping gene *ACT1*. Data are 2^−ΔΔCq^ ± standard deviations; *N* = 3 experiments. NT,  no treatment. (B to D) WT KV1 and Δ*Momca* strains were inoculated as 10-mm mycelial plugs onto 55-mm-diameter plates of complete medium containing H_2_O_2_ or menadione at the concentrations indicated. Images were taken at 7 days after growth. (C and D) Measurements of radial growth of *M. oryzae* WT and mutant strain mycelia under oxidative stress conditions. Results are means from three independent measurements. Error bars denote standard deviations. ns, not significant. Asterisks indicate statistically significant differences (**, *P* < 0.01; ***, *P* < 0.001; ****, *P* < 0.0001; one-way ANOVA with Tukey’s multiple-comparison test using GraphPad Prism 8).

To further verify whether MoMca1 and MoMca2 impact *M. oryzae* survival under oxidative stress as Yca1 does in yeast, we first measured the transcript levels of *MoMca1* and *MoMca2* under different stress conditions. A previous study showed that H_2_O_2_ induces the expression of Yca1, leading to the activation of programmed cell death in yeast (4). Therefore, we used quantitative PCR (qPCR) to analyze the expression of *MoMca1* and *MoMca2* in the WT *M. oryzae* strain under H_2_O_2_ and the free radical generator, menadione. Only *MoMca1* expression was induced in the presence of H_2_O_2_, whereas both metacaspases were induced with menadione, suggesting that the enzymes play distinct roles in responding to cell stress (Fig. 4A).

To measure the sensitivity of the mutants and survival under oxidative stress conditions, we grew the WT and mutant strains on CM plates containing H_2_O_2_ or menadione and compared their radial growth to that of the WT strain. Interestingly, we observed that both the single and double mutants were less sensitive to H_2_O_2_ and menadione conditions than the WT and complemented strains (Fig. 4B). The mutant strains also exhibited higher radial growth under stress than the WT and complemented strains (Fig. 4C and D). These results indicate that the *M. oryzae* metacaspases play an important role in stress-induced programmed cell death.

### Deletion of *MoMca1* and *MoMca2* leads to a delay in conidial germination and appressorium formation.

**FIG 5:**
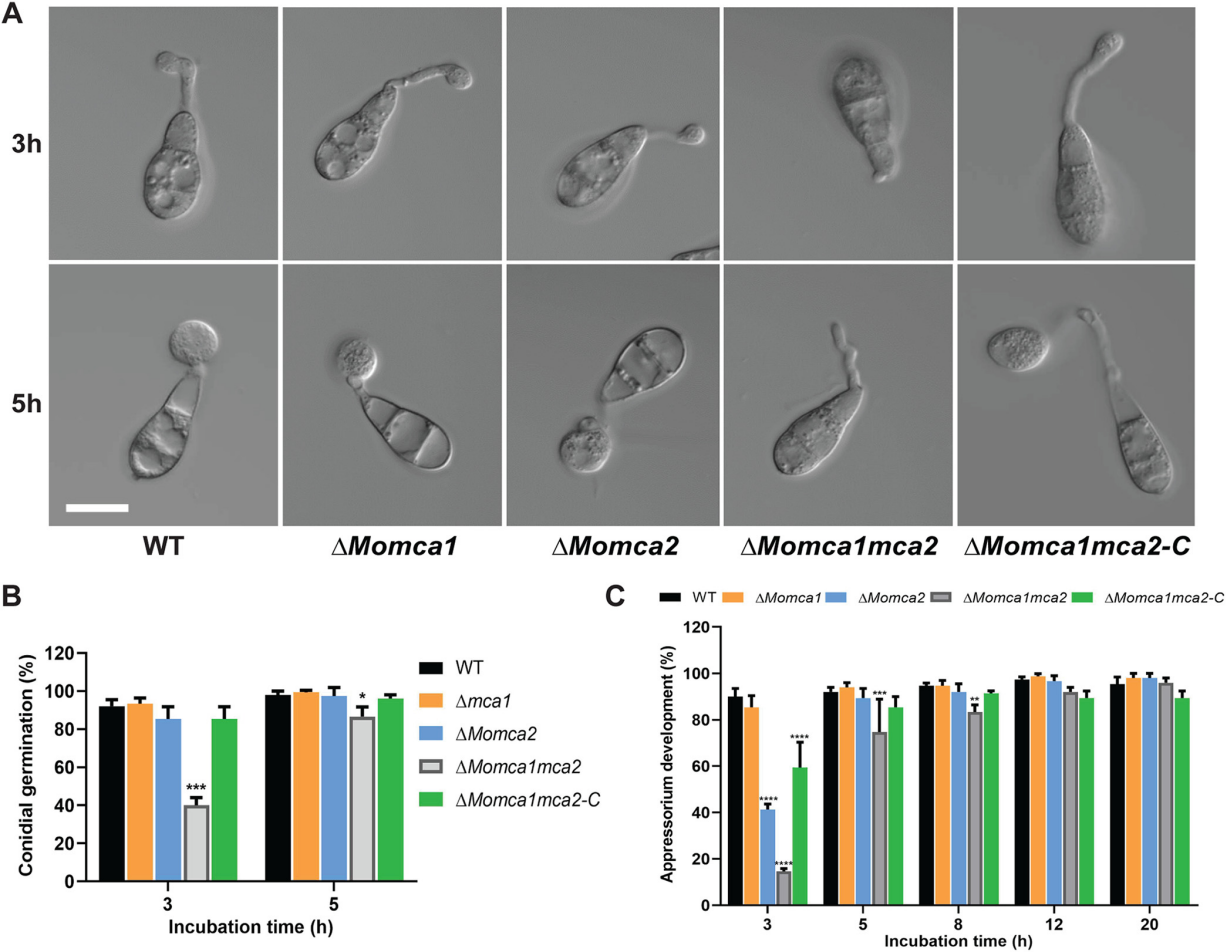
Loss of metacaspase genes delays conidial germination and subsequent formation of appressorium on hydrophobic surfaces. (A) Spores of WT KV1, Δ*Momca1*, Δ*Momca2*, Δ*Momca1mca2*, and Δ*Momca1mca2*-C were applied to artificial hydrophobic surfaces (coverslips). (B) At 3 and 5 h postinoculation (hpi), the rate of conidial germination was measured and compared with that of the WT. (C) At 3, 5, 8, 12, and 20 hpi, the percentage of appressorium development was measured and compared with that of the WT. Results are means from three independent biological measurements. A total of 150 spores were counted for each replicate. Images were taken at each time point. Error bars denote standard deviations. Asterisks indicate statistically significant differences (*, *P* < 0.05; **, *P* < 0.01; ***, *P* < 0.001; ****, *P* < 0.0001; two-way ANOVA with Tukey’s multiple-comparison test using GraphPad Prism 8). Scale bar is 10 μm.

**FIG 6:**
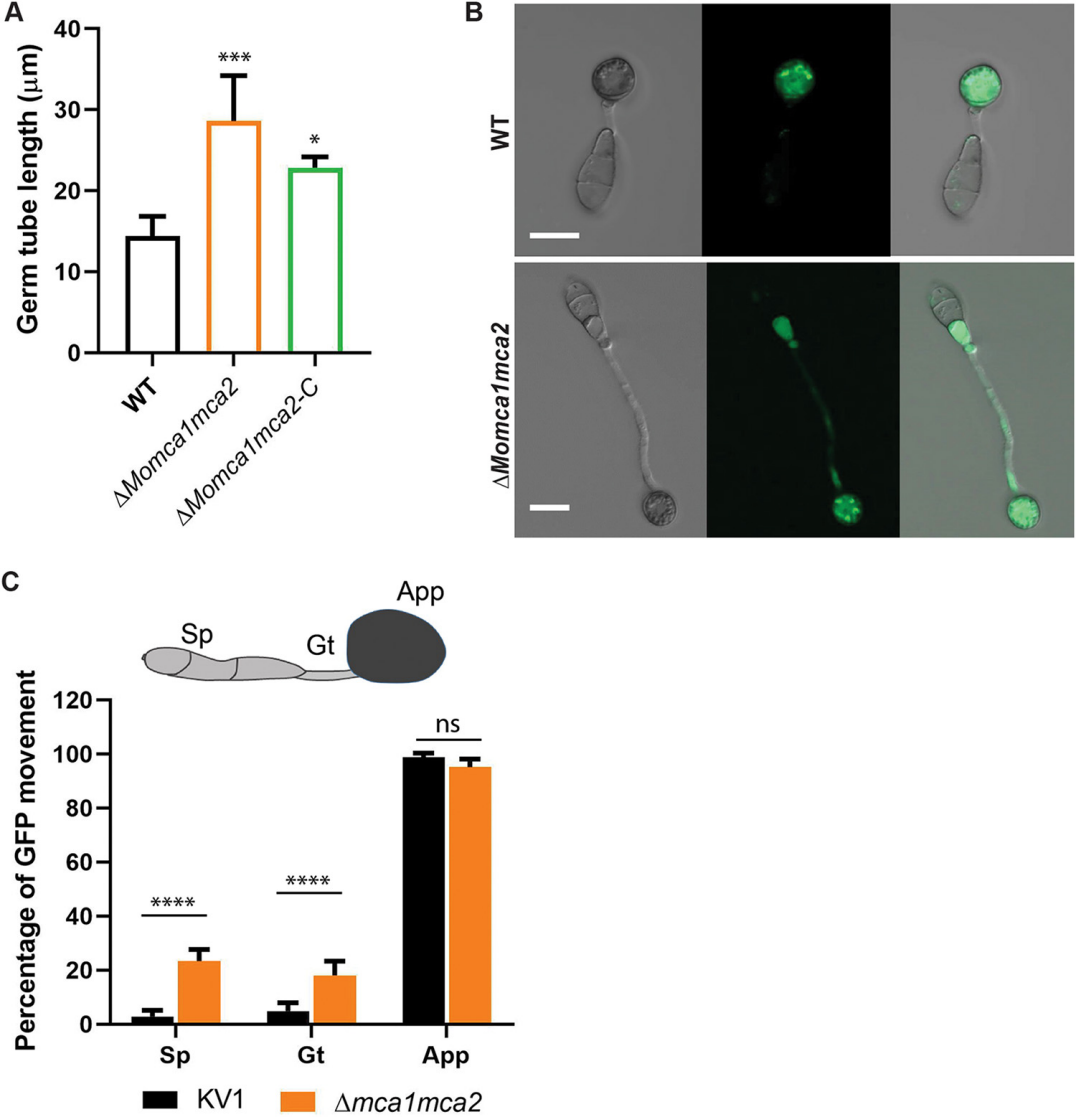
Δ*Momca1mca2* mutant strain developed long germ tubes during appressorium formation. (A) Average germ tube length of the WT, Δ*Momca1mca2*, and Δ*Momca1mca2-C* mutant strains at 20 h postincubation. (B and C) Conidium content at 20 h after incubation on inducible surface. Shown is the percentage of cytoplasmic eGFP movement into the appressorium in the Δ*Momca1mca2* compared to the WT strain. Results are means from three independent measurements. A total of 150 germinated spores with appressoria were counted for each replicate. Sp, spores; Gt, germ tube; App, appressorium. Error bars denote standard deviations. ns, not significant. Asterisks indicate statistically significant differences (*, *P* < 0.05; ***, *P* < 0.001; ****, *P* < 0.0001; one-way ANOVA with Tukey’s multiple-comparison test using GraphPad Prism 8). Scale bar is 10 μm.

Next, we addressed whether *MoMca1* and *MoMca2* play a role in conidial germination or appressorium development. We collected spores from 10-day-old CM plates of the WT and mutant strains and placed spores in suspension on a hydrophobic surface. At 3 and 5 h postincubation (hpi), the WT strain germinated and began to form immature appressoria (Fig. 5A and B). In contrast, the Δ*Momca1mca2* strain was impaired in germination and appressorium morphogenesis (Fig. 5A to C). The Δ*Momca1* and Δ*Momca2* single mutants showed nonsignificant reduction in germination rates. However, the Δ*Momca2* mutant showed a reduction in appressorium development at 3 hpi but appeared similar to the WT at 5 hpi (Fig. 5A to C). At 12 and 20 hpi, all the strains were able to develop appressoria (Fig. 5C). Interestingly, at 20 hpi we observed that approximately 20% of the germinated spores in the Δ*Momca1mca2* strain showed elongated germ tubes compared to those of the WT and single mutants (Fig. 6A and B). These data suggest that *M. oryzae* metacaspases play an important, but redundant, role in conidial germination, as there is a delay in appressorium development in their absence.

### *M. oryzae* metacaspases play a role in the clearance of protein aggregates.

Based on previous studies implicating the role of Yca1 in maintaining protein homeostasis, we next investigated whether the mutants alter protein stability during conidial germination and appressorium formation (6). In *M. oryzae*, the conidium contents are recycled into the appressorium, contributing to the next stages of appressorium development (19, 20). To observe the cytoplasmic protein content during appressorium formation, we used the KV1 strain that harbors a cytosolic enhanced green fluorescence protein (eGFP) reporter protein (23). Spores from WT KV1 and Δ*Momca1mca2* strains were collected and incubated on a hydrophobic surface for 20 hpi. Normally, on hydrophobic surfaces, *M. oryzae* spores germinate and form a short germ tube with an immature appressorium within 6 h. During this process, the conidial contents migrate into the incipient appressorium (19, 24). Using eGFP as a marker, we can observe the movement of proteins into the conidium during this developmental process. For the WT KVI strain, Fig. 6B and C show the expected cytoplasmic movement of proteins from the conidium into the appressorium. In contrast, in the Δ*Momca1mca2* strain, there is a delay of eGFP delivery due to the delay in appressorium formation.

**FIG 7:**
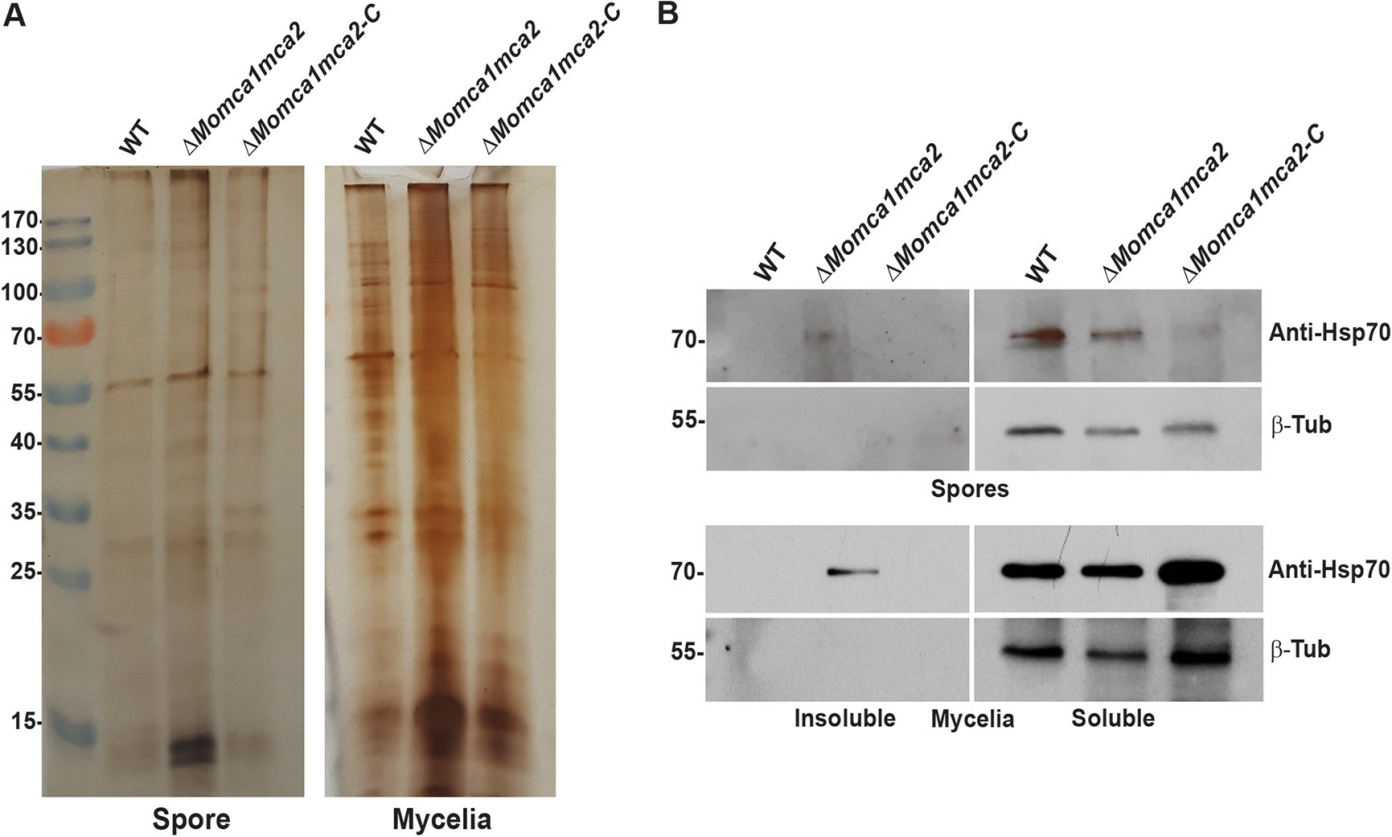
MoMca1 and MoMca2 play an essential role in clearance of insoluble aggregates. (A) Silver-stained 1D PAGE gel of insoluble fractions from equal amounts of total WT KV1, Δ*Momca1mca2*, and Δ*Momca1mca2-C* spores and mycelial lysates. (B) Hsp70 accumulates in the insoluble fractions of Δ*Momca1mca2*’s spores and mycelial lysates. Western blot analysis of Hsp70 in the WT, Δ*Momca1mca2*, and Δ*Momca1mca2-C* strains is shown. Anti-HSP70 was used for protein detection in the insoluble fractions of spores and mycelial lysates. β-Tubulin (β-Tub) was used as a loading control. The experiment was performed three times, and a representative blot is presented.

In yeast, Yca1 plays an essential role in protein quality control by promoting the removal of insoluble protein aggregates to maintain the fitness of new cells (6). The Δ*yca1* mutant showed a significant increase of insoluble aggregates in the cells compared to the WT yeast strain (6). Therefore, we next asked if a similar phenotype could be observed in *M. oryzae*. We measured the insoluble protein aggregate fractions in *M. oryzae* WT, Δ*Momca1mca2* mutant, and the Δ*Momca1mca2-C* complemented strain under normal gel electrophoresis. We observed an increase of insoluble aggregates in the Δ*Momca1mca2* mutant compared to the WT and a restoration of the WT phenotype in the complemented strain (Fig. 7A and Fig. S3C and D). The observations support the hypothesis that *M. oryzae* metacaspases play an important role in preventing protein aggregates from accumulating during appressorium formation. Previous reports demonstrated that the Hsp70s aggregate-remodeling chaperones are overexpressed as a compensatory response to yeast Yca1 deletion (6). To further study the protein aggregation phenotype associated with metacaspase deletion, we assessed the expression of the 70-kDa heat shock protein, Hsp70, in the spores and mycelial lysates. Using Western blot analysis, we detected the expression of Hsp70 in the soluble and insoluble fractions of WT, Δ*Momca1mca2*, and Δ*Momca1mca2-C* strains (Fig. 7B). As Fig. 7 shows, Hsp70 was found to accumulate in the insoluble fraction of the Δ*Momca1mca2* mutant but not the WT and complemented strains. Moreover, these results may lead to a better understanding of metacaspase roles in maintaining protein homeostasis in *M. oryzae* cells throughout development and pathogenesis.

## DISCUSSION

Metacaspases are multifunctional proteases essential for the normal physiology and pathology of nonmetazoan species. These proteases have been extensively characterized in programmed cell death and nonapoptotic processes. Although the metacaspase family exists in eukaryotic organisms, the functional roles of metacaspase proteins have been mostly uncharacterized in pathogenic fungi, especially plant pathogens. This prompted us to investigate the role of metacaspases in the rice blast fungus *M. oryzae*. In this study, we characterized the functions of MoMca1 and MoMca2 metacaspases in *M. oryzae*. Our studies found that *M. oryzae* metacaspases function not only in conidiation and germination but also in the regulation of clearance of insoluble protein aggregates during normal fungal growth.

We bioinformatically identified two type 1 metacaspase proteins, MoMca1 and MoMca2, in the *M. oryzae* genome. These proteins contain an N-terminal prodomain rich in Q/N residues and a C-terminal peptidase C14 caspase domain. MoMca1 and MoMca2 adopt the structure of a caspase fold similar to that of the yeast Yca1 core (see Fig. S1A in the supplemental material).

Due to the bioinformatic similarities of MoMca1 and MoMca2 with Yca1, we looked at the potential link in Ca^2+^ zymogen activation. In contrast to canonical caspases, metacaspases do not undergo dimerization for their activation. Instead, the activity of metacaspases depends on the presence of calcium ions (22, 25), with the only known exception being Arabidopsis thaliana AtMC9, whose activity was shown to be Ca^2+^ independent (5, 26). However, the *Arabidopsis* AtMCP2d metacaspase exhibits a strict Ca^2+^ dependence in millimolar concentrations for its catalytic activation (26). In Yca1, the addition of Ca^2+^ activates the enzyme and leads to autoprocessing events (22). Our biochemical studies have identified MoMca1 and MoMca2 to be Ca^2+^-activated proteases, similar to the Yca1 metacaspase. First, we observed that the autocatalytic processing of MoMca1 and MoMca2 is stimulated by the presence of Ca^2+^ *in vitro*. In addition to the activation requirements for Ca^2+^, we found two cysteine residues that were essential for Yca1 activity were also important for MoMca1 and MoMca2 activity.

Similar to the role of canonical caspases, yeast Yca1 metacaspase positively regulates apoptosis under several stress conditions (27). In this report, we provide evidence that high levels of oxidative stress inducers, such as H_2_O_2_ and menadione, upregulate the expression of *M. oryzae* metacaspase genes. These observations lead us to investigate the functional similarities between *M. oryzae* metacaspases and yeast Yca1. In early studies, Madeo and colleagues demonstrated that low concentrations of H_2_O_2_ facilitate the activation of Yca1 promoting yeast apoptosis (4). Lack of yeast Yca1 activity confers resistance to oxidative stress conditions and a higher survival rate under them (4). Here, we provide evidence that both MoMca1 and MoMca2 can substitute for the Yca1 function in mediating oxidative stress-induced apoptosis in yeast. Although MoMca1 and MoMca2 functionally complement the oxidative stress phenotype in yeast, whether the *M. oryzae* metacaspases respond to similar external stimuli remains unclear. Moreover, the Δ*Momca1mca2* strain shows resistance to H_2_O_2_ and menadione, similar to the Δ*yca1* strain.

Additionally, the deletion of either *MoMca1* or *MoMca2* in *M. oryzae* impairs developmental processes while having no significant reduction in vegetative growth rate on solid media. We demonstrate that both conidiation and symptom development are metacaspase-dependent processes in *M. oryzae*, suggesting a role for these proteins in cell development. The Δ*Momca1mca2* strain was significantly impaired in proper conidiation and pathogenicity.

To be a successful pathogen, *M. oryzae* undergoes several morphological changes to colonize and proliferate inside the rice cells (18). Once the conidium attaches to the leaf cuticle, it germinates and develops a germ tube that will lead to the formation of the melanized appressorium. This morphogenesis process is tightly regulated by the cell cycle checkpoints and occurs within hours (2 to 24 h) after conidium attachment (28). Surprisingly, we discovered that the Δ*Momca1mca2* mutant exhibits delayed conidial germination under inducible conditions, suggesting that these proteins contribute to initial processes that precede appressorium morphogenesis. This delayed phenotype in the Δ*Momca1mca2* mutant disrupts the timing of the appressorium maturation process. Moreover, we observed a delay in the movement of conidial contents into the appressorium. It is well known that during appressorium development, the conidial contents are recycled and delivered to the appressorium, leading to conidial degradation (24). This multistage process is highly regulated by cell cycle progression and autophagy-dependent cell death (24, 28, 29). However, how *M. oryzae* metacaspases cross talk with autophagy processes to regulate appressorium development remains largely unknown.

In other systems, metacaspases display varied roles in pathogenicity and oxidative stress resistance. For instance, the filamentous fungus Podospora anserina contains two metacaspases, and the deletion of both metacaspase genes leads to lower growth rate and fertility, indicating a function in developmental processes (14). However, a single deletion strain, *PaMca1*, has a life span-prolonging effect and resistance response to oxidative stress (14). In contrast, the CasA/CasB metacaspases in the human pathogen Aspergillus fumigatus showed no resistance to oxidative stress or to other apoptosis-induced agents. However, the Δ*casA* Δ*casB* mutant displays a growth defect under endoplasmic reticulum stress conditions, indicating that these proteases confer vital cellular functions under certain stress conditions rather than an involvement in cell death processes (13). Recent studies identified a single metacaspase gene in Ustilago maydis that exhibits impaired growth under either normal growth or oxidative stress conditions (16). The *U. maydis mca1* mutant also displayed a reduction in pathogenicity compared to the wild type, demonstrating that Mca1 in *U. maydis* is required for cellular homeostasis during cell development (16). In this study, we found that the *M. oryzae* Δ*Momca1mca2* mutant exhibited a reduction in pathogenicity on rice plants. With these observations, we can infer that metacaspase functions vary among different organisms and have partial redundancy or antagonistic functions under various settings.

In S. cerevisiae, Yca1 metacaspase appears to be critical for the removal of insoluble protein aggregates during physiological growth conditions. The deletion of *yca1* was associated with an accumulation of insoluble aggregates during logarithmic growth that correlated with an enrichment on vacuolization and stress-response chaperones (6).

Due to previous findings about Yca1 promoting clearance of protein aggregates, we decided to investigate the involvement of our metacaspases in this process. It is noteworthy that in the Δ*Momca1mca2* mutant we observed the accumulation of insoluble protein aggregates during normal growth conditions not present in the control. These findings clearly suggest that *M. oryzae* metacaspases contribute to the removal of insoluble aggregates to maintain optimal fitness during fungal growth. However, we are not clear how *M. oryzae* mediates the clearance of insoluble aggregates during physiological growth. We initially hypothesized that the accumulation of the insoluble aggregates has a direct effect in delaying conidial germination in *M. oryzae*, although a recent study on protein aggregates in *M. oryzae* found no effect on germination when inducing protein aggregates in the spores (30). Although protein aggregates were present in large quantities in catalytic mutants, these spores did not have delayed germination (30). These data suggest that germination itself in the Δ*Momca1mca2* mutant is affected in other ways, independent of protein aggregate accumulation, potentially through some other metacaspase regulatory function. The discovery of metacaspase substrates will give us new insights into the mechanism of activation *in vivo* and the downstream events.

Our work characterizing the role of the *M. oryzae* metacaspases MoMca1 and MoMca2 has demonstrated that biochemically there are several shared properties with the Yca1 metacaspase. MoMca1 and MoMca2 are Ca^2+^-dependent peptidases. While MoMca1 and MoMca2 are dispensable during vegetative growth, they are critical for inducing apoptosis under oxidative stress conditions. More importantly, the deletion of these genes leads to an accumulation of protein aggregates. The most striking phenotype of the *M. oryzae* metacaspases is that they are critical for sporulation and, subsequently, pathogenicity in rice blast disease progression.

## MATERIALS AND METHODS

### Strains and culture conditions.

The *M. oryzae* strain KV1 was used as the wild-type strain throughout this research (23) (see Table S1 in the supplemental material). This strain and its transformants were cultured on complete medium (CM; 1% [wt/vol] glucose, 0.2% [wt/vol] peptone, 0.1% [wt/vol] yeast extract, 0.1% [wt/vol] Casamino Acids, 0.1% trace elements, 0.1% vitamin solution, and 1× nitrate salts) and incubated at 25°C under 12-h light/dark cycles for 5 to 12 days. For DNA and RNA extraction, all strains were grown on CM liquid medium at 25°C with agitation for 48 h. For sporulation rates, strains were grown on at least three independent CM plates. After 12 days of growth, spores were harvested and counted using a hemocytometer (Corning). To observe vegetative mycelial growth under stress, 10 mM H_2_O_2_ and 150 μM menadione were individually added to CM agar medium. A mycelial disc (3.5 mm in diameter) of each strain was inoculated on stress medium containing either H_2_O_2_ or menadione, and the growth rate was assessed by measuring culture diameters after 5 days of growth (unless otherwise stated). Plate images were taken with an Epson perfection V700 photo scanner. For routine cloning, the Escherichia coli DH5α strain was grown in 2×YT broth at 37°C. The Saccharomyces cerevisiae yeast strain BY4741 was grown in synthetic dropout (SD) medium as described previously (31) at 30°C.

10.1128/mBio.03471-20.4 TABLE S1 Strains used in this study. Download Table S1, PDF file, 0.1 MB. Copyright © 2021 Fernandez et al. 2021 Fernandez et al. This content is distributed under the terms of the Creative Commons Attribution 4.0 International license.

### Generation of plasmids.

The *M. oryzae Mca1* and *Mca2* coding sequences were amplified by PCR using *M. oryzae* cDNA as a template. For *in vitro* transcription/translation reactions, *S. cerevisae Yca1*, *M. oryzae Mca1*, and *Mca2* coding sequences were cloned into pET15b bacterial expression plasmid containing an N-terminal 6×His tag. Single-amino-acid mutations were introduced via QuikChange site-directed mutagenesis.

For yeast expression, *MoMca1* and *MoMca2*, full-length and variant versions, were cloned into the XhoI/HindIII sites on a galactose-driven yeast expression vector, pESC-Leu (kind gift from Vincent S. Tagliabracci).

To test the redundancy of *M. oryzae* Mca1 and Mca2 to the yeast metacaspase Yca1, we obtained a Yca1 knockout yeast strain (*yca1*Δ::KANMX; Dharmacon) derived from strain BY4741 (32).

### Sequence analysis.

The *MoMca1* and *MoMca2* coding gene sequences were obtained from the Magnaporthe oryzae database, http://fungi.ensembl.org/Magnaporthe_oryzae/Info/Index. Metacaspase sequences were collected using the MoMca1 (NCBI accession no. A4QTY2.2) sequence as a query for PSI-BLAST (33) (5 iterations, E value cutoff of 0.005) against a sequence database containing the RefSeq representative prokaryotic genome set (1,684 genomes, from 28 August 2018) and latest representative eukaryotic genomes (from 28 August 2018). Collected sequences (2,712) were clustered using CLANS (34) and colored according to taxonomy. A multiple-sequence alignment of representative sequences was generated using MAFFT (35) and colored according to conservation: mainly hydrophobic (yellow), mainly small (gray), active site (black), and calcium activation (red). To identify a structure template for MoMca1, the sequence (A4QTY2.2) was submitted to the HHPRED server (36) to search against the PDB database, which confidently identified the Yca1 structure (PDB entry 4F6O) as a top template (100% probability; score, 2.2e−38). The HHPRED pairwise alignment between MoMca1 and Yca1 was used to generate a structure model for MoMca1 using SwissModel (37).

### Fungal transformation and complementation.

Targeted gene replacements for *MoMca1* and *MoMca2* were carried out using the PCR-based split marker method (38) in which the *HPH* gene (1.4 kb), conferring resistance to hygromycin, and *ILV1* gene (2.8 kb), conferring resistance to sulfonylurea, replaced the coding sequence of each gene. Approximately 1 kb upstream and downstream of the gene was used for homologous recombination. The flanking sequences (∼1 kb) of the *MoMca1* and *MoMca2* genes were amplified and fused to the selectable marker by PCR (Fig. S1A). PCR products were transformed into *M. oryzae* WT protoplasts. Positive transformants carrying homologous gene replacement of the gene of interest were confirmed by PCR using the nested primers shown in Table S2.

10.1128/mBio.03471-20.5 TABLE S2 Oligonucleotide primers used in this study. Download Table S2, PDF file, 0.01 MB. Copyright © 2021 Fernandez et al. 2021 Fernandez et al. This content is distributed under the terms of the Creative Commons Attribution 4.0 International license.

To generate the complemented strain, full-length *MoMca1* and *MoMca2* genes, including their native promoter, were amplified and inserted into pBGt (39) fungal expression vector by using the Gibson Assembly approach (NEB BioLabs). The pBGt-MoMca1Mca2 expression vector was transformed into Δ*Momca1mca2* protoplasts and selected on gentamicin-containing plates. The complementation strain was identified by PCR (Table S2). Single-spore isolation was performed for all strains.

### Spore germination and appressorium development assays.

Spores were harvested from 10-day-old cultures, filtered through two layers of Miracloth (Millipore Sigma), and resuspended to a concentration of 5 × 10^4^ spores/ml in sterile water. For conidial germination, 20 μl of conidial suspension was placed on glass coverslips (Fisher Scientific, St. Louis, MO, USA) and incubated at 24°C in a humid chamber. The percentages of conidial germination and appressorium formation were assessed at 3, 5, 8, 12, and 20 h postincubation. Average mean values were determined from 150 conidia and performed in three independent experiments. Germ tube lengths were measured using ImageJ. All imaging was performed on a Zeiss LSM 800 confocal microscope, and images were converted using ImageJ (NIH).

### Pathogenicity assays.

Spores from the WT and transformants were harvested from 10-day-old cultures on 0.2% gelatin as previously described (40). Three-week-old rice seedlings (YT16) were spray inoculated with 2 × 10^4^ spores/ml conidial suspension. Inoculated plants were placed in a humidity chamber for 24 h and then transferred back to the growth chamber with 12-h light/dark cycles. Disease severity was assessed at 7 days postinoculation. Photographs of diseased rice leaves were taken. The number of pixels under lesion areas and healthy areas of diseased leaves were calculated by using ImageJ.

### Yeast transformation.

Yeast transformations were performed using the lithium acetate (LiAc) method as described previously (31). Briefly, yeast cells were grown overnight at 30°C in yeast extract-peptone-dextrose (YPD) medium. The overnight cultures were diluted in 50 ml of fresh YPD (adjusted to an optical density at 600 nm [OD_600_] of 0.1) and then cultured until reaching an OD of 0.5 to 0.7. After incubation, the yeast cells were collected by centrifugation and washed twice with solution 1 (1× Tris-EDTA [TE], 1 M lithium acetate, pH 7.5, with acetic acid). The pellet was resuspended with 500 μl of solution 1. In 100 μl of yeast cells, 1 μg of DNA, 5 μl DNA carrier, and 5 μl of 100% dimethyl sulfoxide (DMSO) were added and gently mixed. A volume of 700 μl of solution 2 (solution 1 supplemented with 50% polyethylene glycol) was added and incubated at 30°C for 30 min. The yeast cells were heat shocked at 42°C for 15 min and washed with TE, pH 7.5. Samples were plated on SD medium minus leucine or uracil and incubated at 30°C for 2 to 3 days.

### Survival assay.

Yeast survival assay was performed as described previously (4). Briefly, yeast strains harboring the expression vector were grown overnight in 3 ml liquid SD-Leu glucose medium at 30°C. The overnight cultures were diluted in 10 ml of fresh medium (adjusted to an OD_600_ of 0.05) and then cultured until reaching an OD_600_ of 0.4 to 0.6. The yeast cells were collected and resuspended in SD-Leu supplemented with 2% galactose and 1% raffinose. Cell counts were equalized based on OD_600_ before treatment. For stimulation of yeast apoptosis, cells were treated with the final concentration of 1.2 mM H_2_O_2_ and incubated for 24 h at 30°C. An aliquot of each culture was 10-fold diluted in sterile water and plated in SD glucose plates. Colonies were counted after 2 to 3 days, and treated samples were compared with nontreated samples to calculate survival rate. This experiment was conducted in biological triplicates.

### RNA extraction, cDNA synthesis, and qRT-PCR analysis.

To quantify metacaspase expression under oxidative stress conditions, the fungal strains were grown on liquid CM for 48 h at 25°C with agitation (200 rpm) before treatment with or without 10 mM H_2_O_2_ and 150 μM menadione for 2 h. Mycelia were harvested and ground with a pestle and mortar in liquid nitrogen and stored at −80°C. A total of 100 mg of ground mycelia was used to perform RNA extractions. Total RNA was isolated using the RNeasy plant mini plus kit from Qiagen. RNA concentration was measured via NanoDrop, and cDNA was generated using the iScript cDNA synthesis kit (Quanta). Primers were designed to amplify 100 to 150 bp of each target gene (Table S2) and tested for efficiency. Transcripts were quantified on a CFX384 Touch real-time PCR detection system using the iTaq universal SYBR green supermix (Quanta) and 500 nM primers. Relative gene expression for each target gene was calculated by the −ΔΔ*C_T_* method. The expression of each gene was normalized against the *M. oryzae* actin gene (*ACT1*). Results are given as the averages from three technical replications and three biological replications.

### *In vitro* transcription/translation and purification.

Coupled transcription/translation reactions were carried out per the manufacturer’s instructions (L1170; Promega). Briefly, rabbit reticulocyte lysate was incubated with 1 μg of the appropriate expression plasmid and 2 ml of [^35^S]l-methionine (1,000 Ci/mMol at 10 mCi/ml) (NEG709A; Perkin Elmer) for 90 min at room temperature. Reaction mixtures were diluted in 1 ml of 50 mM Tris-HCl (pH 7.5), 10 mM NaCl, and 1 mM dithiothreitol (DTT). A volume of 100 μl of DEAE-Sepharose (DFF100; Millipore Sigma) slurry was added to the reaction mixtures and incubated on a nutator for 30 min at room temperature. The slurry was washed three times with 1 ml of 50 mM Tris-HCl (pH 7.5), 10 mM NaCl, and 1 mM DTT and eluted with 500 μl of 50 mM Tris-HCl (pH 7.5), 500 mM NaCl, and 1 mM DTT. Eluents were collected and incubated with 50 μl of packed nickel-nitrilotriacetic acid resin (His-Pur 88222; Bio-Rad) at room temperature with nutating for 30 min. The beads were washed three times with 1 ml of 50 mM Tris-HCl (pH 7.5), 150 mM NaCl, 20 mM imidazole, and 1 mM DTT. Proteins were eluted using 100 μl of 50 mM Tris-HCl (pH 7.5), 150 mM NaCl, 300 mM imidazole, and 1 mM DTT.

### Protease assays.

Protease reactions were carried out in a buffer solution containing 50 mM Tris-HCl (pH 7.5), 150 mM NaCl, 1 mM DTT, with or without 1 mM CaCl_2_. Reaction mixtures were incubated at 30°C overnight and stopped by the addition of Laemmli buffer containing β-mercaptoethanol and boiling. Reaction products were resolved on a 12% polyacrylamide gel by electrophoresis and autoradiography.

### Protein aggregate assays.

Protein fractions from spores and mycelial cell lysates were collected as described previously (6), with some modifications. Briefly, spores were harvested from 10-day-old cultures, filtered through two layers of Miracloth (Millipore Sigma), and resuspended to a concentration of 5 × 10^5^ spores/ml in sterile water. Spores were recovered by centrifugation, and pellets were frozen down and stored at −80°C for later processing. Mycelium was harvested and ground with a pestle and mortar in liquid nitrogen and stored at −80°C. A total of 100 mg of ground mycelia was used to performed protein extractions. Pellet samples were thawed in equal volumes of lysis buffer (0.1% Triton X-100, 50 mM Tris, pH 7.4, 1 mM EDTA, and 1% glycerol supplemented with 5 mM Na_3_VO_4_ and 1 mM phenylmethylsulfonyl fluoride) and acid-washed glass beads (Sigma-Aldrich). Samples were vortexed for 20 min with 1-min on/off cycles. To remove the cell debris, samples were centrifuged at 2,000 rpm. The total cell lysate was collected and protein concentration was measured and normalized. The cell lysate was centrifuged at 15,000 × *g* for 15 min at 4°C to separate the soluble (supernatant) and insoluble (pellet) fractions. Pellets were washed twice with lysis buffer containing 10% Triton X-100. Pellets were treated with 4 M urea and boiled in 2× Laemmli sample buffer for 5 min. Proteins were loaded on 12.5% SDS-polyacrylamide gel electrophoresis for one-dimensional (1D) PAGE separation for silver staining or transferred to polyvinylidene difluoride membranes. HSP70 expression was detected with mouse anti-HSP70 (1:5,000; Abcam) antibody, and β-tubulin (polyclonal anti-β-TUB; Santa Cruz) was used as a loading control.

### Statistical analysis.

All data are given as means ± standard deviations from at least three independent experiments (unless stated otherwise). Each experiment was conducted in triplicate. Statistical analyses were performed by using one-way analysis of variance (ANOVA) and Tukey’s multiple-comparison test. A *P* value of <0.05 was considered significant.
