# Supplementary figures and images for: Role of Two Metacaspases in Development and Pathogenicity of the Rice Blast Fungus Magnaporthe oryzae
Source: mBio. 2021 Feb 9;12(1):e03471-20. doi: 10.1128/mBio.03471-20 (PMC7885106; doi:10.1128/mBio.03471-20)

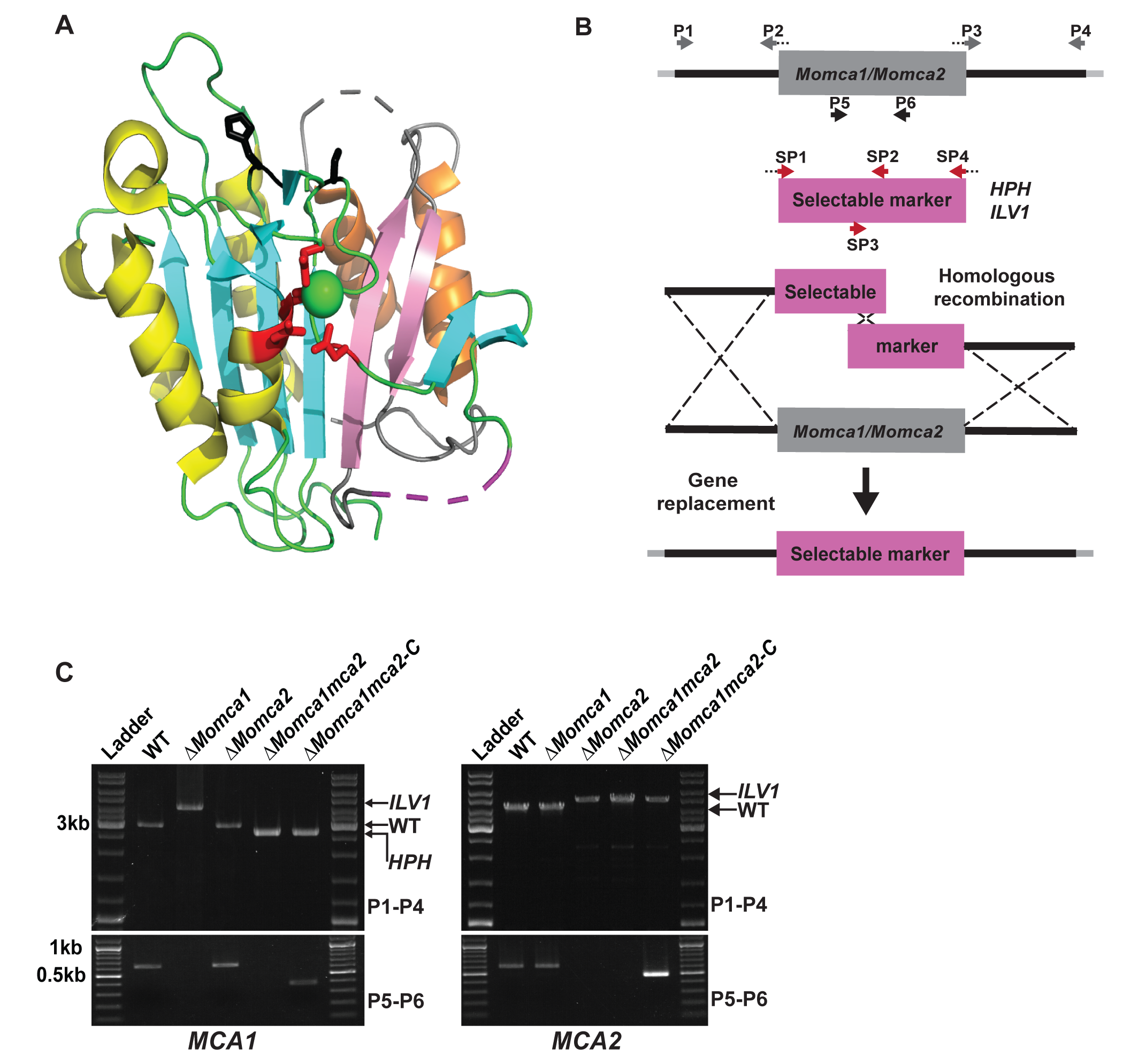

Supplement: FIG S1 [file mBio.03471-20-sf001.tif]

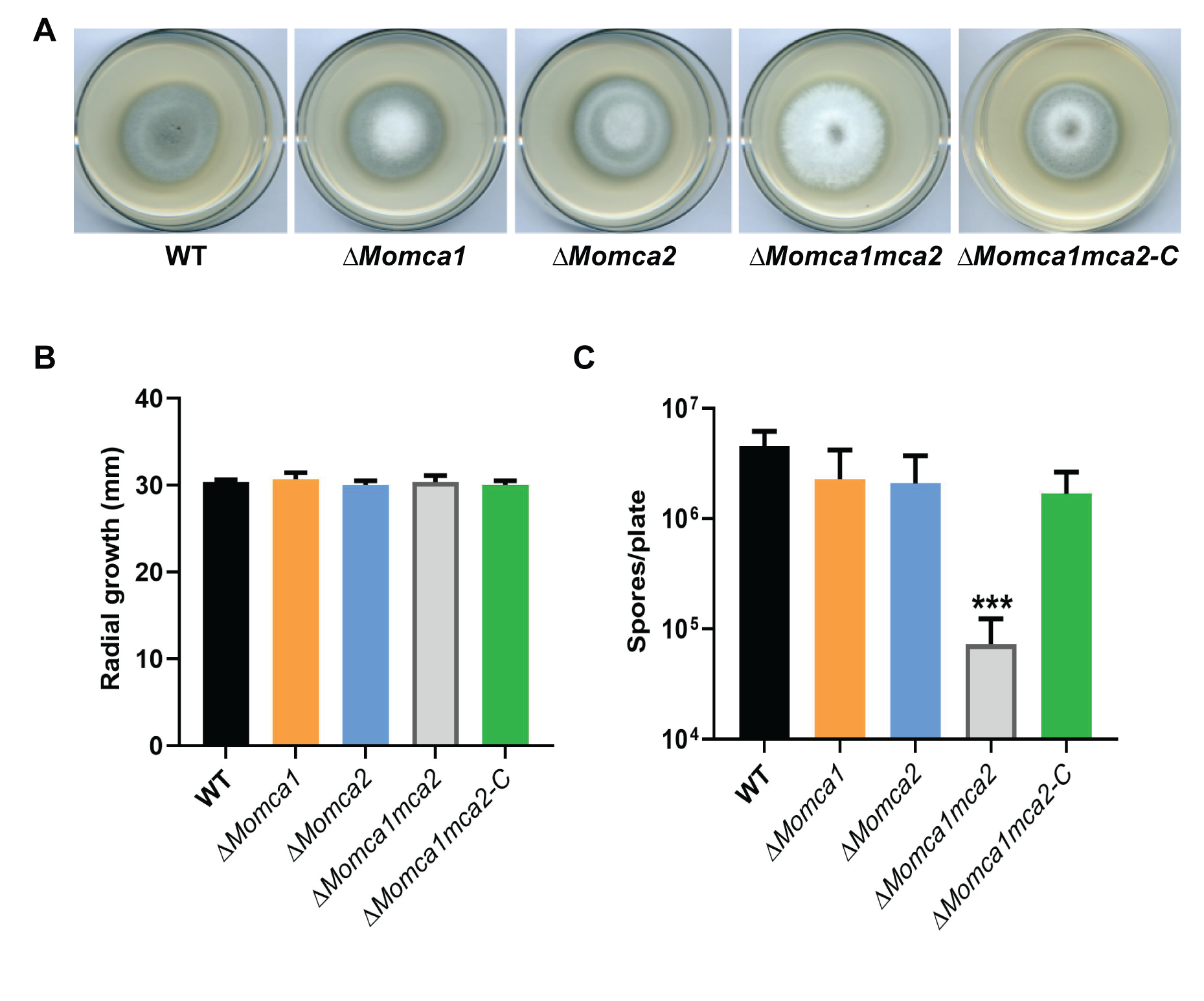

Supplement: FIG S2 [file mBio.03471-20-sf002.tif]

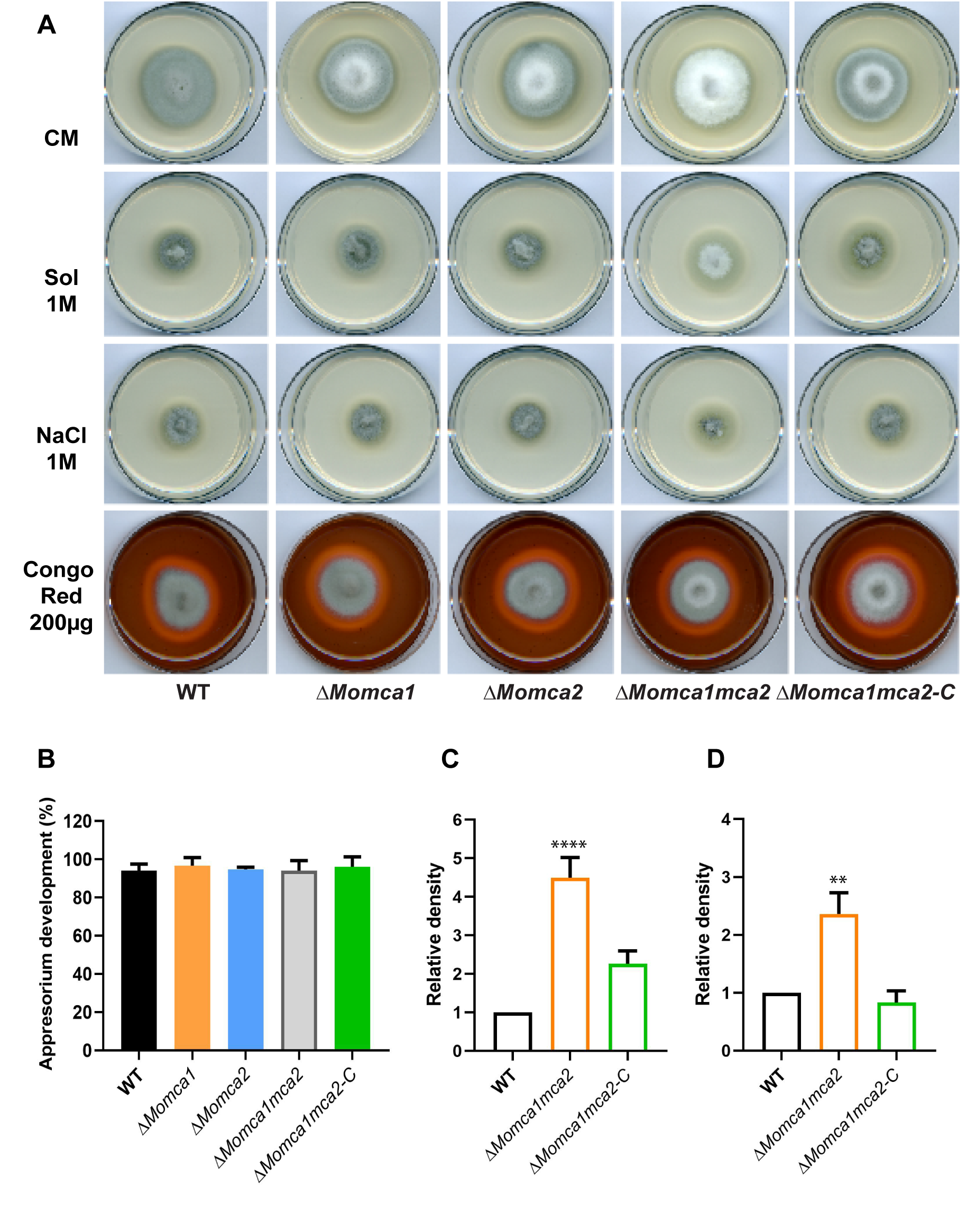

Supplement: FIG S3 [file mBio.03471-20-sf003.tif]
