# Supplementary material for: Role of Two Metacaspases in Development and Pathogenicity of the Rice Blast Fungus Magnaporthe oryzae
Source: mBio. 2021 Feb 9;12(1):e03471-20. doi: 10.1128/mBio.03471-20 (PMC7885106; doi:10.1128/mBio.03471-20)
Supplement: TABLE S1 [file mBio.03471-20-st001.pdf]

Table S1. *Magnaporthe oryzae* strains used in this study.

Strain	Genotype	Reference
KV1	O-137 with cytoplasmic eGFP	(24)
Δ*Momca1*	MGG_04926 deletion mutant of KV1	This study
Δ*Momca2*	MGG_13530 deletion mutant of KV1	This study
Δ*Momca1mca2*	MGG_04926 and MGG_13530 deletion mutant of KV1	This study
Δ*Momca1mca2-C*	Complementation strain with pBGt carrying the full-length *MoMCA1 and MoMCA2* genes and their native promoters	This study
E. coli (DH5α)	pGB1805-yca1 ORF (YOR197W)	Dharmacon, Inc
*yca1*Δ	MATa *yca1*::KanR *his3*Δ*0 leu2*Δ*0 met15*Δ*0 ura3*Δ*0*	Dharmacon, Inc
BY4741	MATa *his3*Δ*0 leu2*Δ*0 met15*Δ*0 ura3*Δ*0*	(33)
*yca1*Δ::Yca1	MATa yca1::KanR *his3*Δ*0 leu2*Δ*0 met15*Δ*0 ura3*Δ*0* pESC-LEU-Yca1	This study
*yca1*Δ::MoMca1	MATa yca1::KanR *his3*Δ*0 leu2*Δ*0 met15*Δ*0 ura3*Δ*0* pESC-LEU-MoMca1	This study
*yca1*Δ::MoMca2	MATa yca1::KanR *his3*Δ*0 leu2*Δ*0 met15*Δ*0 ura3*Δ*0* pESC-LEU-MoMca2	This study
