# Supplementary material for: Role of Two Metacaspases in Development and Pathogenicity of the Rice Blast Fungus Magnaporthe oryzae
Source: mBio. 2021 Feb 9;12(1):e03471-20. doi: 10.1128/mBio.03471-20 (PMC7885106; doi:10.1128/mBio.03471-20)
Supplement: TABLE S2 [file mBio.03471-20-st002.pdf]

Table S2. Oligonucleotide primers used in this study.

Gene	Primer	Sequence 5' – 3'
**Split marker gene deletion**		
ILV1<br>Sulfonylurea resistance	M13F:IL	**CGCCAGGGGTTTTCCCAGTCACGAC**<br>GTCGACGTGCCAACGCCACAG
	ILSplit	AAGCATGTGCAGTGCCTTC
	M13R:LV1	**AGCGGATAACAATTTCACACAGGA**TCGACGTGAGAGCATGCTAA
	LV1Split	GAGGCCGACGTCATAGGCATC
HPH<br>Hygromycin resistance	M13F:HY	**CGCCAGGGGTTTTCCCAGTCACGAC** CTGATATTGAAGGAGCATT
	HYSplitR	GGATGCCTCCGCTCGAAGTA
	M13R:YG	**AGCGGATAACAATTTCACACAGGA** CTATTCCTTTGCCCTCGGACGA
	YGSplitF	CGTTGCAAGACCTGCCTGAA
MoMca1;<br>MGG_04926	LF-FW	ATAGATGAAGTGATAGTAGCAGGTGAG
	LF-REV	**GTCGTGACTGGGAAAACCCTGGCG**<br>GTGAAGGTGTGGATATCTCAGATAT
	RF-FW	**TCCTGTGTGAAATTGTTATCCGCT**<br>CTACACTCTACCAACAGACTACGGAT
	RF-REV	GTCTGATTAGAACATGATTAGTCTCTTG
	NesFW	CCTTGTCAGATTATGGTCGAGG
	NesREV	TTGCATTTTATAGAGAAG
MoMca2;<br>MGG_13530	LF-FW	GCTAAGACACTCGACCCAAGTGC
	LF-REV	**GTCGTGACTGGGAAAACCCTGGCG**<br>CAAGGTGGAAATGTTGCGTATAAG
	RF-FW	**TCCTGTGTGAAATTGTTATCCGCT**<br>CCGCAGCTGAGACCTATATACTCTCT
	RF-REV	ATAACCACAGTGCTGAGCGTCG
	NesFW	AGGACTAGCGGTGCCGTTTATC
	NesREV	TCTCAGAATAAGCCGACCCTTTGT
**Gene specific primers for qPCR**		
MoMca1	*qMoMca2-F*	CAGGAACCAGACTTACAACCAG
	qMoMca2-R	CTGAGGTTTCTGGCTGTACTG
MoMca2	*qMoMca1-F*	AACAAAAGACCTCGACGGAG
	qMoMca1-R	ATGTACGGCAAATCTAGGGC
MoAct1;<br>MGG_03982	*qMoAct1-F*	ACAATGGTTCGGGTATGTGC
	qMoAct1-R	CGACAATGGACGGGAAGAC
**Protein expression**		
pET15b<br>*MoMca1* Nde1<br>FW		GATCCATATGATGAGCGGTTATCCCGGTCA
pET15b<br>*MoMca1* XhoI<br>REV		GATCCTCGAGTTA CATGACAAACCACACGTTTTCG
pET15b<br>*MoMca2* Nde1<br>FW		GATCCATATGATGTCTGGATACCCGGGTCAG
pET15b		GATCCTCGAGTTACAAGATGAACCTTAGATCCG

MoMca2 XhoI REV		
pET15b; *Yca1* NdeI FW		GATCCATATGATGTATCCAGGTAGTGGACGT
pET15b; *Yca1* XhoI REV		GATCCTCGAGCTACATAATAAATTGCAGATTTACG
**Missense mutation**		
pET15b; Yca1 C276A FW		CAGCATTGTTTGACTCTGCTCATTCGGGTACAGTGT
pET15b; Yca1 C276A REV		ACACTGTACCCGAATGAGCAGAGTCAAACAATGCTG
pET15b; MoMca1 C242A FW		GCCATCTTTGACTCTGCTCACTCTGGCACTGC
pET15b; MoMca1 C242A REV		GCAGTGCCAGAGTGAGCAGAGTCAAAGATGGC
pET15b; MoMca2 C257A FW		ATCTTCGACTCCGCCCATTCGGGAAC
pET15b; MoMca2 C257A REV		GTTCCCGAATGGGCGGAGTCGAAGAT
**Complementation**		
pBGt *MoMca1, MoMca2*	FW	ACAGCTATGACATGATTACGAATTCGTGGGTAGCTGCAACCTAG
	REV	TTACTTATCGTCGTCATCCTTGTAATCCATGACAAACCACACGTTTTC
	FW	GATTACAAGGATGACGACGATAAGTAATGAGACTGCGGTTCGGAC
	REV	TAAAACGACGGCCAGTGCCAAGCTTTTAGAGGTCTTCTTCGGAAATCA ACTTCTGTTCCAA GATGAACCTTAGATCCGTG
**Yeast expression**		
pESC-Leu *MoMca1* XhoI FW		GATCCTCGAGATGAGCGGTTATCCCGGTCAAG
pESC-Leu *MoMca1* HindII REV		CCTAAAGCTTTTACATGACAAACCACACGTTTTCGGTA
pESC-Leu *MoMca2* XhoI FW		GATCCTCGAGATGTCTGGATACCCGGGTCAGG
pESC-Leu *MoMca2* HindII REV		CCTAAAGCTTTTA CAAGATGAACCTTAGATCCGTGTTCAG
pESC-Leu *Yca1* XhoI FW		GATCCTCGAGATGTATCCAGGTAGTGGACGT
pESC-Leu *Yca1* HindII REV		CCTAAAGCTTCTACATAATAAATTGCAGATTTACG

2

3
